# Hodgkin's lymphoma presenting with heart failure: a case report

**DOI:** 10.1186/1752-1947-4-14

**Published:** 2010-01-20

**Authors:** Zeinab Amirimoghaddam, Malihe Khoddami, Nahid Dehghan Nayeri, Somayeh Molaee

**Affiliations:** 1 Imam Hosein Hospital, Shahid Beheshti University of Medical Sciences, Shahid Madani St, Nezamabad, Imam Hosein Sq, Tehran 1617763141, Iran; 2 Faculty of Nursing and Midwifery, Tehran University of Medical Sciences, Nosrat St, Tohid Square, Tehran, 1419733171, Iran

## Abstract

**Introduction:**

Cardiac involvement in malignant lymphoma is one of the least investigated subjects in oncology. This article reports a case of cardiac involvement in Hodgkin's lymphoma which presented as heart failure.

**Case presentation:**

We report the case of an 8-year-old Afghan girl with Hodgkin's lymphoma. The disease presented with systemic signs and symptoms, including abdominal distension, weakness, pallor, chills, fever, generalized edema, hepatosplenomegaly and generalized lymphadenopathy, as well as signs of heart failure. Test results showed a rare form of heart metastasis.

**Conclusion:**

We report a case of Hodgkin's lymphoma with metastasis to the heart, detected premortem. Although the involvement of the heart in a malignancy is relatively common, premortem detection is unusual and only few studies have reported it in the literature.

## Introduction

Cardiac involvement in malignant lymphoma is one of the least investigated subjects in oncology [1]. Cardiac metastases are found in 20% to 25% of patients with lymphoma [2,3]. Studies by Roberts *et al*. and Cains *et al*. reported that 9% of all cardiac tumors are related to lymphoma [3,4]. Some authors have described primary cardiac lymphomas presenting with pericardial effusion [5], arrhythmias and heart failure [6]. Echocardiography is known to be a sensitive method for the diagnosis of cardiac involvement in patients with lymphoma. The pattern of cardiac involvement varies with different types of lymphoma, suggesting that different pathologic types of lymphoma may have different mechanisms of metastasis to the heart. Diffused myocardial infiltration documented by echocardiography has rarely been described as a presenting feature of this condition [7,8], but it is commonly found postmortem [9].

Bashir *et al*. explained the pathogenesis of cardiac involvement in lymphoma in their 2006 study [10]. One sixth of neoplastic pericardial diseases are caused by hematological malignancies. However, the incidence, clinical course and outcome of pericardial involvement in Hodgkin's lymphoma are unknown. Lymphomas involve the pericardium mostly via lymphatic or hematogenous metastasis. This type of pericardial involvement generally results in pericardial effusion as a consequence of the obstruction of the venous and lymphatic flows of pericardial fluid. Although most cases are clinically silent, effusions can impair cardiac function. In severe cases it can even lead to pericardial tamponade, which is a life-threatening condition.

The diagnostic standard in these conditions includes acquiring the patient's history and performing physical examinations, chest radiography, contrast-enhanced computed tomography (CT) of the neck, chest, abdomen, and pelvis, and gallium scan or positron emission tomography. Patients with suspected bone involvement usually undergo bone scans, while patients in the advanced stages of the disease undergo bone marrow biopsies. Baseline studies consist of a complete blood count, blood chemistry analyses, kidney and liver function tests, echocardiography, electrocardiography, and pulmonary function tests [10].

Low-dose radiation plus multi-agent chemotherapy for pediatric Hodgkin's disease was adopted after a pioneering study reported using 15 to 25 Gy and six cycles of mechlorethamine, vincristine, procarbazine, and prednisone chemotherapy for children [11]. Chemotherapy standards in Hodgkin's are VEPA (vinblastin, etoposide, prednisolone, adriamycin), VAMP (vinblastin, Adriamycin, methotrexate, prednisone), COP (cyclophosphamide, vincristine, procarbazine) and Stanford V (doxorubicin, vinblastin, mechlorethamine, vincristine, bleomycin, etoposide, prednisone).

Primary presentations of Hodgkin's lymphoma have been reported many times, but heart failure in Hodgkin's lymphoma has not yet been reported in the literature. This case report describes a rare case of cardiac involvement in Hodgkin's lymphoma which presented as heart failure and was diagnosed through echocardiography findings.

## Case presentation

An 8-year-old Afghan girl presented with a two-month history of edema, abdominal distension, weakness, pallor, chills, fever, anorexia, and weight loss. Her medical history was not remarkable. Physical examinations showed severe mucosal and conjunctival pallor, periorbital and sacral edemas, and abdominal distension. She also presented with tender mobile lymph nodes in her right neck (5 × 5 mm), bilateral inguinal area (0.5 cm × 0.5 cm) and left axillary (0.7 cm × 0.7 cm), as well as marked hepatosplenomegaly and ascites with shifting dullness. Systolic murmurs (II/III) of the heart and lungs were apparent.

In this patient, Hodgkin's lymphoma had metastasized to the myocardial tissue. The tumor involved all the cardiac tissue and the septum. Metastasis must have occurred via the blood vessels because it involved the cardiac tissue itself as well as the lymph nodes. Other hematopoetic areas such as the liver, spleen and bone marrow were also involved.

The symptoms of lymphadenopathy included enlargement of the lymph nodes, in particular the para-aortic lymph nodes. There were symptoms of cardiac failure in the form of tachycardia, cardiomegaly, gallop rhythm, tachypnea, weak pulse, and hypotension.

In examining the patient, we used all diagnostic standards except positron emission tomography, because the patient had already been diagnosed with lymphoma and metastasis. There were no signs of Hodgkin's lymphoma in the bone marrow and bone aspiration test results.

Laboratory findings included severe anemia with moderate anisopoikilocytosis, hemoglobin level of 3.2 (normal range 260 to 400 mg/dl), erythrocyte sedimentation rate of 50 (normal range <15 mm/hr), and positive C-reactive protein. Polymerase chain reaction for tuberculosis, blood culture, urine culture, hydatid antibody, Coombs Wright and 2 ME, direct Coombs, bone marrow culture, and blood smears for malaria and borrelia were all negative. Our patient's G6PD level was also normal.

There were several findings that led to the identification of appropriate treatment for our patient. In abdominal sonography, her liver was found to be enlarged with heterogenic echo. Marked hepatosplenomegaly (spans = 17 cm) and two round hypoechoic areas in the hepatic portal space due to adenopathy were seen. Her biliary gall bladder had no stones and its wall had an increased thickness. An abdominal CT scan (with and without contrast) showed severe hepatosplenomegaly, a hypodense area in the liver that might be a small hemangioma or cyst, considerable para-aortic adenopathy, and dilated small bowel loops with thickened walls. Her spleen was enlarged to a diameter of 13 cm and had homogenous echo. Her internal and external biliary tract liver were of normal diameter. There were some circular hypoechoic masses in our patient's portohepatic region, which indicated lymphadenopathy in this area. Her para-aortic region could not been observed because of the abdominal gas. Her intestinal loops were dilated in the pelvic region and were full of liquid.

Her kidneys had normal secretions and appeared normal. No other abnormalities were seen. Her lungs were clear and of normal size, as shown on contrast chest X-ray. The chest X-ray also showed cardiomegaly. A CT scan of our patient's chest showed multiple lymph adenopathies in the paratracheal and subcarinal regions.

Our patient's lung parenchymas were reported to be normal in a thorax CT scan with contrast. There were no effects of impressed masses, parenchymal nodules or abnormal infiltration. The vessels and bronchus seemed normal. Multiple lymph nodes were seen in the para-aorta, subcarina, lungs or esophagus.

Anemia due to tumor involved all parts of the hematopoetic areas such as the liver, bone marrow and spleen, and also due to cardiac deficiency and endocarditis. Cardiac failure could occur after a bacterial endocarditis and the tumor development. There was also lymphadenopathy, pericardial effusion, fever, tremor, and edema.

In treating our patient, acute symptoms such as severe anemia, infection, electrolyte and biomedical imbalances and hypoglycemia were encountered. Once these had been treated, we addressed the Hodgkin's lymphoma.

Our patient was given a high-protein and high-calorie diet, and treatment for tuberculosis was started. Gentamycin, penicillin, and vancomycin were prescribed because of the presenting endocarditis. After stabling the patient's condition, 14 sessions of chemotherapy were started. Chemotherapy included intravenous Adriamycin (doxorubicin) 25 mg/m^2^, Bleomycin 10 mg/m^2^, and vincristine 6 mg/m^2^, as well as 375 mg of dimethyl, triazeno, imidazole and carboxamide (DTIC) as infusion.

After one year, no evidence of the disease were reported in a thorax CT scan with injection contrast or in abdominal and pelvic CT scans with oral and injection contrast. There was also no evidence of abnormal opacity in the lung parenchyma. The pathologic area was not seen either in the thorax bone structure or the adjacent soft tissue. There were no symptoms of either pleural fluid or pleural thickness. There was also no evidence of the anterior, medial of posterior mediastinal masses. The major vessels appeared normal. Small aortocaval and thorocaval lymph nodes were observed. A myeloma with a maximum diameter of 1 cm at the left kidney was detected. The size and density of our patient's kidneys were normal. The urine tract was not obstructed. The hilar areas were normal in both kidneys. The bronchus had normal calibers. The gall bladder and the internal and external liver biliary tracts were also normal. The spleen was of normal size and had systematic margins and homogenous density. The attenuation valve was also normal.

After completing the treatment, our patient was discharged. Her parents were told that she should attend our clinic for follow up every three months. All the symptoms of the disease have now disappeared and the girl is living an ordinary life. She does not have symptoms of lymphadenopathy, splenomegaly or any other problems.

## Conclusion

Although the involvement of the heart in malignancies is relatively common, premortem detection is unusual and only a few studies have reported this subject in the literature. We report a case of Hodgkin's lymphoma which presented with systemic signs and symptoms including abdominal distension, weakness, pallor, chills, fever, generalized edema, hepatosplenomegaly and generalized lymphadenopathy, as well as signs of heart failure. Echocardiography revealed pericardial effusion, left ventricular hypertrophy, and lucent myocardial lesions. A right cervical lymph node biopsy established the diagnosis of nodular sclerosing Hodgkin's lymphoma with involvement of the bone marrow at biopsy. After 14 chemotherapy sessions, systemic and cardiac abnormalities had improved. To the best of our knowledge, this is the first reported case of Hodgkin's lymphoma accompanied by cardiac metastasis and heart failure.

Echocardiograph findings have shown that pericardial effusion is the most common abnormality in cardiac metastasis. The early detection of metastatic cardiac involvement can be beneficial for the patients because it can lead to careful monitoring, better management of morbidity and decreased mortality.

Studies have shown that patients with lymphoma associated with cardiac involvement can be treated successfully.

## Consent

Written informed consent was obtained from the patient's next-of-kin for publication of this case report and any accompanying images. A copy of the written consent is available for review by the Editor-in-Chief of this journal.

## Competing interests

The authors declare that they have no competing interests.

## Authors' contributions

ZA and MK gathered and interpreted data on the patient. NDN and SM assisted in gathering and organizing the data. They were also major contributors in writing the manuscript. All authors read and approved the final manuscript.
